# Calcific Plug Obstructing the Thoracic Aorta

**DOI:** 10.1055/s-0039-1701000

**Published:** 2020-06-29

**Authors:** Federico Del Re, Andrea De Martino, Giosuè Falcetta, Uberto Bortolotti

**Affiliations:** 1 Section of Cardiac Surgery, Department of Cardiac Thoracic and Vascular Surgery, University Hospital, Pisa, Italy

**Keywords:** aortic occlusion, hypertension, aortic syndrome

## Abstract

We describe a case of a 57-year-old woman with occlusion of the thoracic aorta due to a calcific plug as an unusual cause of refractory hypertension. Middle aortic syndrome, also known as “ *coral reef aorta* ,” defined as a clinical condition caused by thoracic and/or abdominal aortic stenosis, requires peculiar treatment strategies representing a complex surgical challenge.

Middle aortic syndrome is defined as a clinical condition caused by thoracic and/or abdominal aortic stenosis. 1 Congenital coarctation, Takayasu or giant cell arteritis, neurofibromatosis, and Williams syndrome represent the most frequent etiologies.

We describe a 57-year-old woman with refractory hypertension of the upper body as initial presentation of mid-aortic syndrome due to an extensive calcified plug causing occlusion of the descending thoracic aorta.

The patient was referred to our hospital for pulmonary edema due to a hypertensive crisis. Her past history included a subarachnoid hemorrhage successfully treated by clipping of a saccular aneurysm of the right internal carotid artery. The electrocardiogram showed signs of left ventricular hypertrophy and the chest X-ray evidenced moderate cardiac enlargement. Arterial blood pressure in the upper extremities was 200 mm Hg systolic but only 80 mm Hg in the lower limbs, with an ankle/brachial index of 0.4. An echocardiography-Doppler study showed >70% stenosis at the origin of both renal arteries.

Computed tomography evidenced diffuse calcification involving the entire aorta with a calcific plug creating obstruction of the descending thoracic aorta below the origin of the left subclavian artery ( Fig. 1 ).

**Fig. 1:**
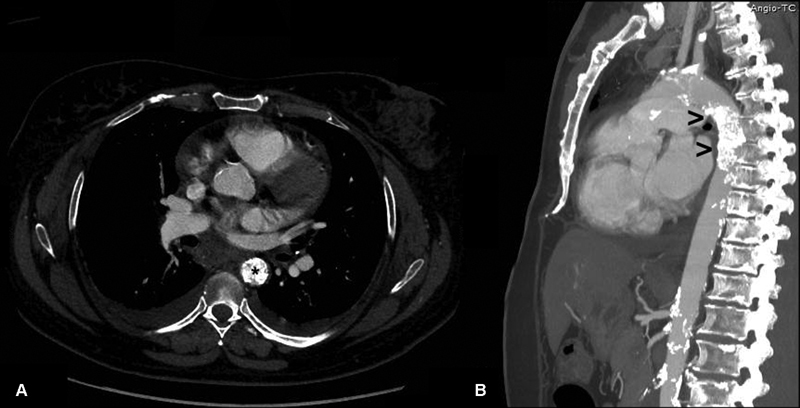
Computed tomography showing occlusion of the descending thoracic aorta (axial view in panel **A** and sagittal view in Panel **B** ) by a massive calcific plug (asterisk in Panel **A** and arrowheads in Panel **B** ). Diffuse calcification of the entire aorta is also present.

Endovascular treatment of the occluded aorta along with direct surgical repair due to the extensive calcifications of the aortic wall was considered too hazardous.

In this case severe hypertension was considered due to a combination of aortic occlusion and bilateral stenosis of the renal arteries. In the absence of symptoms related to abdominal and lower limb ischemia, endovascular treatment of renal artery stenosis has been scheduled. Angioplasty of the renal arteries was not performed since the patient died 6 months later because of acute bowel infarction.

Alternative surgical procedures, in case of ischemic symptoms, may include an apicoaortic conduit or an axillobifemoral bypass.

This case describes an uncommon cause of middle aortic syndrome, also known as “ *coral reef aorta* ” which can represent an extremely difficult surgical challenge. In adult patients with refractory arterial hypertension and ankle-brachial index suggestive for aortic obstruction, a complete tomographic evaluation of the aorta is mandatory. 2
